# High prevalence of respiratory diseases: a population-based ecological study, Sertão do Araripe, 2008-2019

**DOI:** 10.1590/S2237-96222025v34e20240519.en

**Published:** 2025-09-15

**Authors:** José Rafael Soares da Silva, Rodrigo Gomes de Arruda, Bárbara de Oliveira Silva, Erika da Silva Bezerra de Menezes, Cinthia Martins Menino Diniz, João Pedro Alves Gomes, Breno Caldas de Araújo, Moacyr Jesus Barreto de Melo Rêgo, Maira Galdino da Rocha Pitta, Michelly Cristiny Pereira

**Affiliations:** 1 Universidade Federal de Pernambuco, Núcleo de Pesquisa em Inovação Terapêutica Suely Galdino, Recife, PE, Brazil; 2 Universidade Federal de Pernambuco, Departamento de Economia, Recife, PE, Brazil

**Keywords:** Respiratory Tract Diseases, Pneumonia, Occupational Health, Mortality, Prevalence, Enfermedades Respiratorias, Neumonía, Salud Laboral, Mortalidad, Prevalencia

## Abstract

**Objective:**

To investigate the prevalence and mortality of respiratory diseases between 2008 and 2019 in municipalities with gypsum industry activity in the Sertão do Araripe region, Pernambuco, Brazil.

**Methods:**

This was a population-based ecological study using data extracted from hospital and mortality information systems. Ten municipalities from the gypsum production hub were compared to other municipalities in Pernambuco that do not have gypsum industry activity. Prevalence and mortality rates were calculated per 100,000 and 1,000 inhabitants and presented by mean values (standard deviation) for chronic obstructive pulmonary disease, asthma, acute bronchitis, and pneumonia. Pearson’s correlation tests, Mann-Whitney U tests, and multiple linear regression were performed to assess the relationship between gypsum industry activity and respiratory diseases.

**Results:**

During the study period, 17,630 cases of respiratory diseases were recorded, with a mean prevalence of 454.74 (147.16) per 100,000 inhabitants and a mortality rate of 0.34 (0.08) per 1,000 inhabitants. Pneumonia accounted for 12,680 hospitalizations, with a prevalence of 431.20 (173.04) per 100,000 inhabitants, being most frequent in Ouricuri (543.08), Ipubi (409.93), and Moreilândia (404.80). A higher prevalence of pneumonia was observed in municipalities within the gypsum production hub (p-value 0.007). A positive correlation was found between respiratory diseases and the hospital bed occupancy rate (r 0.24; p-value<0.050), and a negative correlation with per capita public health expenditures. There was a significant association between the presence of the gypsum industry and pneumonia (p-value<0.001).

**Conclusion:**

The gypsum industry’s activity is associated with the prevalence of respiratory diseases in the region, with a particular emphasis on the high rates of pneumonia.

## Resumen

**Objetivo:**

Investigar la prevalencia y la mortalidad por enfermedades respiratorias, entre 2008 y 2019, en los municipios con actividad yesera en la región del Sertão do Araripe, Pernambuco.

**Métodos:**

Se trata de un estudio ecológico, basado en la población, con datos extraídos de los sistemas de información hospitalaria y de mortalidad, comparando 10 municipios del polo de gesso con el resto de municipios de Pernambuco sin actividad de extracción de gesso. Las tasas de prevalencia y mortalidad se calcularon por cada 100 000 y 1000 habitantes y se presentaron como media (desviación estándar) para la enfermedad pulmonar obstructiva crónica, el asma, la bronquitis aguda y la neumonía. Se realizaron pruebas de correlación de Pearson, U de Mann-Whitney y regresión lineal múltiple para evaluar la relación entre la actividad yesera y las enfermedades respiratorias.

**Resultados:**

En el período analizado, se registraron 17 630 casos de enfermedades respiratorias, con una prevalencia media de 454,74 (147,16) por cada 100 000 habitantes y una mortalidad de 0,34 (0,08) por cada 1000 habitantes. La neumonía fue responsable de 12 680 hospitalizaciones, con una prevalencia de 431,20 (173,04) por cada 100 000 habitantes, siendo más frecuente en Ouricuri (543,08), Ipubi (409,93) y Moreilândia (404,80). Se observó una mayor prevalencia de neumonía en los municipios del polo yesero (valor p 0,007). Se verificó una correlación positiva entre las enfermedades respiratorias y la tasa de ocupación de camas hospitalarias (r 0,24; valor p<0,050), y negativa con el gasto público en salud per cápita. Hubo una asociación significativa entre la presencia de la industria del yeso y la neumonía (valor p<0,001).

**Conclusión:**

La actividad del yeso está asociada a la prevalencia de enfermedades respiratorias en la región, destacando los elevados índices de neumonía.

**Ethical aspects**

This research used public domain and anonymized databases.

## Introduction

The state of Pernambuco holds approximately 18.0% of Brazil’s gypsum reserves and accounts for 97.0% of the country’s total production of this mineral (1). Extraction and processing activities are predominantly concentrated in the Sertão do Araripe region, encompassing the municipalities of Araripina, Bodocó, Exu, Granito, Ipubi, Moreilândia, Ouricuri, Santa Cruz, Santa Filomena, and Trindade (2).

The gypsum industry plays a key role in regional economic growth, as it promotes employment and contributes to the region’s socioeconomic development. However, the calcination and extraction processes involved in gypsum production pose potential risks to the respiratory health of the local population (3). Prolonged exposure to agents present in mining or construction companies, such as silica (gypsum dust) and smoke (from biomass burning), may increase the risk of inflammatory and infectious lung processes (4), in addition to a range of respiratory diseases such as silicosis (5), asthma (6), bronchitis, pneumonia (7), chronic obstructive pulmonary disease (8), pneumoconiosis (7,9), and lung cancer (10). 

Due to the limited number of studies addressing the epidemiological aspects of respiratory diseases in the Sertão do Araripe region and the need to implement effective public health policies, this study aimed to investigate the prevalence and mortality of respiratory diseases, from 2008 to 2019, in municipalities with gypsum industry activity in that region.

## Methods

### Study design and size

This population-based ecological study was conducted from 2008 to 2019. It analyzed data from 185 municipalities in the state of Pernambuco, including 10 municipalities from the Sertão do Araripe region and the remaining 175 municipalities, as well as the Fernando de Noronha archipelago. The study included data on hospitalizations and deaths due to respiratory diseases associated with exposure to gypsum dust, including pneumoconiosis, acute bronchitis, pneumonia, asthma, and chronic obstructive pulmonary disease. These outcomes affected both the general population and the economically active population (individuals aged 15–59 years).

### Context and bias control

The selected period was defined to include only data prior to the COVID-19 pandemic. Data after this period were excluded due to the exponential increase in cases and deaths caused by the novel coronavirus in Brazil from 2020 onwards (11), which could result in bias in the analyses performed.

### Setting and study population

According to the 2022 Demographic Census, Pernambuco had an estimated population of 9,058,931 inhabitants and a population density of 92.37 inhabitants per square kilometer. The state ranks 19th in Brazil in terms of land area, with a territory of 98,067.877 km^2^. In 2021, it had the 15th lowest Human Development Index in the country, below the national average (12).

Hospitalization and mortality data were selected as comparative parameters between the previously mentioned regions.

### Study variables

The independent variables selected for this study were: presence of a gypsum production hub, population size, gross domestic product (GDP) per capita (adjusted to 2010 prices), per capita public health expenditures, and hospital bed rate. 

Population size and gross domestic product per capita (adjusted to 2010 prices) data were obtained from the Brazilian Institute of Geography and Statistics (available from: https://cidades.ibge.gov.br/). Data on public health expenditures per capita and hospital bed rates were obtained from the Hospital Information System of the Department of Informatics of the Brazilian National Health System (available from: https://datasus.saude.gov.br/acesso-a-informacao/morbidade-hospitalar-do-sus-sih-sus/). These data were analyzed to evaluate possible correlations and effects on the prevalence rates of respiratory diseases. 

### Information systems used 

Data on hospital morbidity were analyzed using the Hospital Information System and Mortality Information System, available from the Department of Informatics of the Brazilian National Health System, based on place of residence in Pernambuco (available from: http://datasus.saude.gov.br/acesso-a-informacao/morbidade-hospitalar-do-sus-sih-sus/). Prevalence and mortality rates were calculated based on population data from the Brazilian Institute of Geography and Statistics, using 2022 Census data (available from: https://www.ibge.gov.br/). 

Confirmation of economic activities related to gypsum production was obtained from the Brazilian Annual Social Information Report platform for the period 2008–2019 (available from: https://bi.mte.gov.br/bgcaged/). The analysis considered the following subclasses of the Brazilian National Classification of Economic Activities 2.0: (i) extraction of gypsum and kaolin, including the mining of raw materials for production; (ii) manufacture of gypsum products and similar construction materials; (iii) manufacture of lime and gypsum, including industrial calcination processes; and (iv) gypsum finishing and stucco work, representing specialized services in the application of coatings and moldings in buildings. This study did not require approval by a research ethics committee, as it used publicly available data.

### Statistical analysis

Data were organized in Microsoft Excel 2010 spreadsheets and analyzed using STATA 17.0 MP software. Descriptive statistics, such as means, medians, and standard deviations, were calculated, and boxplot diagrams were constructed to illustrate the distribution of prevalence and mortality due to respiratory diseases. The comparison of prevalence rates across different age groups in municipalities of Pernambuco was performed using the Mann-Whitney U test, which is appropriate for comparing data distributions without assuming a specific probability distribution.

Temporal trends in prevalence rates were analyzed using repeated measures analysis of variance (ANOVA), with F-test calculation, allowing comparison of rate evolution between municipalities with and without gypsum industry activity. Pearson’s correlation was performed to identify associations between respiratory diseases, socioeconomic variables, and health infrastructure through bivariate analysis. Subsequently, a multiple linear regression model was estimated using the ordinary least squares method, with forward variable selection (13,14), and represented by the following equation: (disease rate)= B^0^+B^1^ (gypsum hub)+ B^2^ (population size)+ B^3^ (gross domestic product per capita)+ B^4^ (public health expenditures per capita)+ B^5^ (hospital bed rate)+ε.

The coefficient of determination (R^2^) was used to measure the explained variability, and residual analysis assessed the model’s adequacy (Supplementary Figure 1).

**Figure 1:**
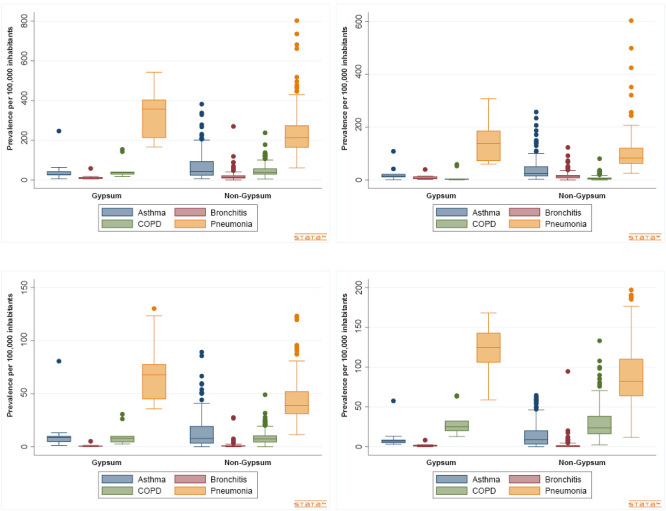
Median prevalence of respiratory diseases, with data for the general population (A), children under 14 years of age (B), economically active population aged 15-59 years (C), and older adults aged 60 years or older (D), in the 10 municipalities of the Sertão do Araripe region compared to the 175 municipalities of Pernambuco, 2008-2019, with 430,305 cases included

Analyses focused on the 10 municipalities of the Sertão do Araripe region, given the region’s importance to the gypsum industry. To test the robustness of the effects, independent variables were introduced individually in separate models, allowing assessment of the stability and statistical significance of the coefficients, particularly the variable representing the presence of the gypsum production hub. The thematic prevalence maps were created using QGIS 3.30.2. It utilized seven-color gradients to represent the spatial distribution of respiratory diseases in the analyzed municipalities.

### Data access and cleaning methods

Hospitalization and death records related to respiratory diseases were extracted from the databases, including pneumoconiosis (International Statistical Classification of Diseases and Related Health Problems [ICD] J63), acute bronchitis (ICD J20), pneumonia (ICD J18), asthma (ICD J45), and chronic obstructive pulmonary disease (ICD J44), all associated with occupational and environmental exposure to gypsum dust (7). Additionally, we analyzed whether there were significant differences in the rates of these diseases among the economically active population (individuals aged 15–59 years) residing in the 10 municipalities of the Sertão do Araripe compared to the rates in the other 175 municipalities. 

### Data matching

Matching was performed based on socioeconomic variables, including population size, gross domestic product per capita, public health expenditure per capita, and hospital bed rate for the 185 municipalities of Pernambuco. These variables were controlled to ensure that the differences observed in the prevalence and mortality rates for respiratory diseases were mainly attributed to the presence of the gypsum hub. A statistical difference was considered when the p-value was less than 0.05.

## Results

The Sertão do Araripe region recorded 17,630 cases of respiratory diseases, including asthma, acute bronchitis, chronic obstructive pulmonary disease, and pneumonia between 2008 and 2019. The mean prevalence was 454.74 (147.16) cases per 100,000 inhabitants, with children aged 1–4 years presenting the highest mean of 984.04 (384.14) cases, followed by older adults over 80 years of age, with a mean of 882.95 (270.15) cases. The mean mortality rate was 0.34 (0.08) cases per 1,000 inhabitants.

There were 1,474 asthma-related hospitalizations in the Sertão do Araripe region, with a mean (standard deviation) prevalence of 52.26 (70.06) cases per 100,000 inhabitants. The highest prevalence rates occurred between 2008 and 2010. The municipalities with the highest rates were Moreilândia, Ipubi, and Granito, with rates of 246.67; 63.77, and 44.25, respectively. The most affected groups were children aged 1–4 years, with a mean of 124 (122.15) cases; children aged 5–9 years, with a mean of 98.38 (135.95) cases; and older adults aged 60–69 years, with a mean of 66.83 (117.46) cases. The highest mortality rates per 1,000 inhabitants were observed in Granito, Exu, and Trindade (0.02; 0.01, and 0.01, respectively).

For acute bronchitis, the mean prevalence in the region was 14.20 (16.02) cases per 100,000 inhabitants. The highest rates were reported in Araripina, Ipubi, and Ouricuri, with 58.17; 15.70, and 12.51 cases per 100,000 inhabitants, respectively. The year 2011 presented the highest regional mean prevalence, with 24.54 cases per 100,000 inhabitants. The age groups most affected by the disease, according to prevalence calculations, were infants aged 0-1 year, with 72.77 (105.66) cases, children aged 1-4 years, with 39.81 (76.86) cases, and children aged 5-9 years, with 10.47 (31.42) cases. Only Araripina and Ouricuri recorded deaths, with a mortality rate of less than 0.01 cases per 1,000 inhabitants in both municipalities.

There were 2,578 hospitalizations related to chronic obstructive pulmonary disease, with a mean prevalence for the region of 55.11 (49.72) cases per 100,000 inhabitants. There have been intermittent increases and declines over the years, with Araripina and Granito being the municipalities with the highest incidence: 153.95 (92.33) and 142.33 (168.99) cases, respectively. The most affected age group in the region was individuals aged 70-79 years, with 123.68 (76.97) cases per 100,000 inhabitants. The highest average mortality rates from chronic obstructive pulmonary disease were found in Moreilândia (0.158 cases per 1,000 inhabitants), Santa Filomena (0.11 cases per 1,000 inhabitants), and Bodocó (0.11 cases per 1,000 inhabitants).

There were 12,680 hospitalizations due to pneumonia in the region. Prevalence peaked in 2011, with 451.155 (294.70) cases per 100,000 inhabitants, decreased in 2013, and increased again in 2017 and 2019, with 389.26 (103.11) and 431.20 (173.04) cases per 100,000 inhabitants, respectively. Pneumonia was the only disease with a significantly higher prevalence in the ten municipalities of the Sertão do Araripe compared to the 175 municipalities outside the gypsum production hub in Pernambuco. The highest prevalence rates were observed in Ouricuri, 543.08 (267.11), Ipubi, 543.08 (267.11), and Moreilândia, 404.80 (305.01). Children aged 1–4 years had the highest prevalence rates, with 758.46 (394.31) cases per 100,000 inhabitants, followed by adults over 80 years of age, with 697.61 (246.46) cases per 100,000 inhabitants. The highest pneumonia-related mortality rates were observed in Bodocó, Exu, and Moreilândia, with 0.26; 0.25, and 0.25 cases per 1,000 inhabitants, respectively.

No statistically significant differences were observed in prevalence and mortality among the economically active population when each disease was analyzed individually, except for pneumonia. Regarding pneumonia, the median prevalence was significantly higher in municipalities within the gypsum-producing region compared to those in the non-gypsum-producing region (Figure 1).

The median in the gypsum hub was 357.20 (n=10), compared to 213.20 (n=175) outside the hub, with a statistically significant difference (U 1,571.0; p-value 0.007). Among the population under 14 years of age, pneumonia prevalence was also higher in municipalities within the gypsum hub: 138.70 (n=10), compared to a median of 83.08 outside the hub, with evidence of statistical significance (U 1,580.0; p-value 0.040). For the economically active population (15–59 years), prevalence was higher in the gypsum hub (median 67.87) than outside the hub (median 38.90; U 1,575.0; p-value<0.001). Regarding older adults, prevalence was also higher in the gypsum hub (median 124.4) compared to the non-hub municipalities (median 82.18; U 1,387.0; p-value 0.014). Hospitalizations due to pneumoconiosis were rare, with only one case in Araripina and another in Bodocó; no deaths were recorded.

**Figure 2:**
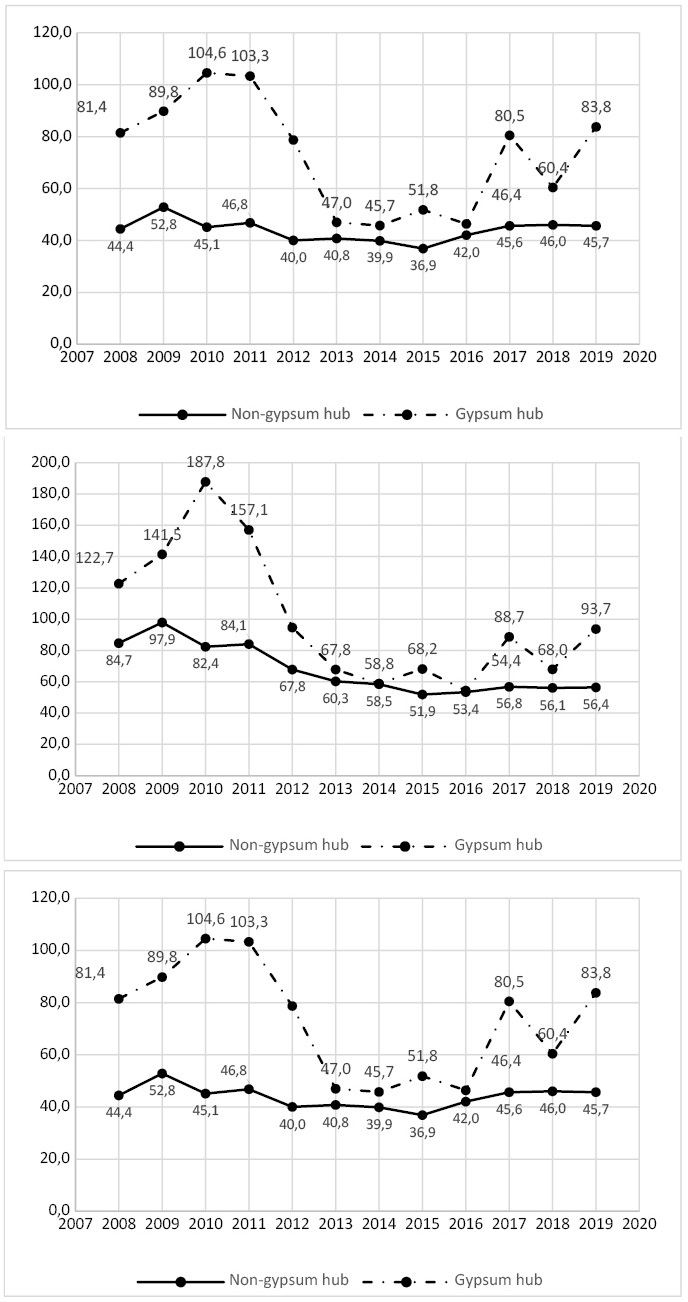
Mean prevalence for respiratory diseases, including asthma, acute bronchitis, chronic obstructive pulmonary disease, and pneumonia, for the general population (A), the economically active population aged 15-59 with respiratory diseases (B) and the economically active population aged 15-59 with pneumonia (C), in the 10 municipalities of the Sertão do Araripe and in the remaining 175 municipalities of Pernambuco, 2008-2019, with 430,305 cases included

**Figure 3:**
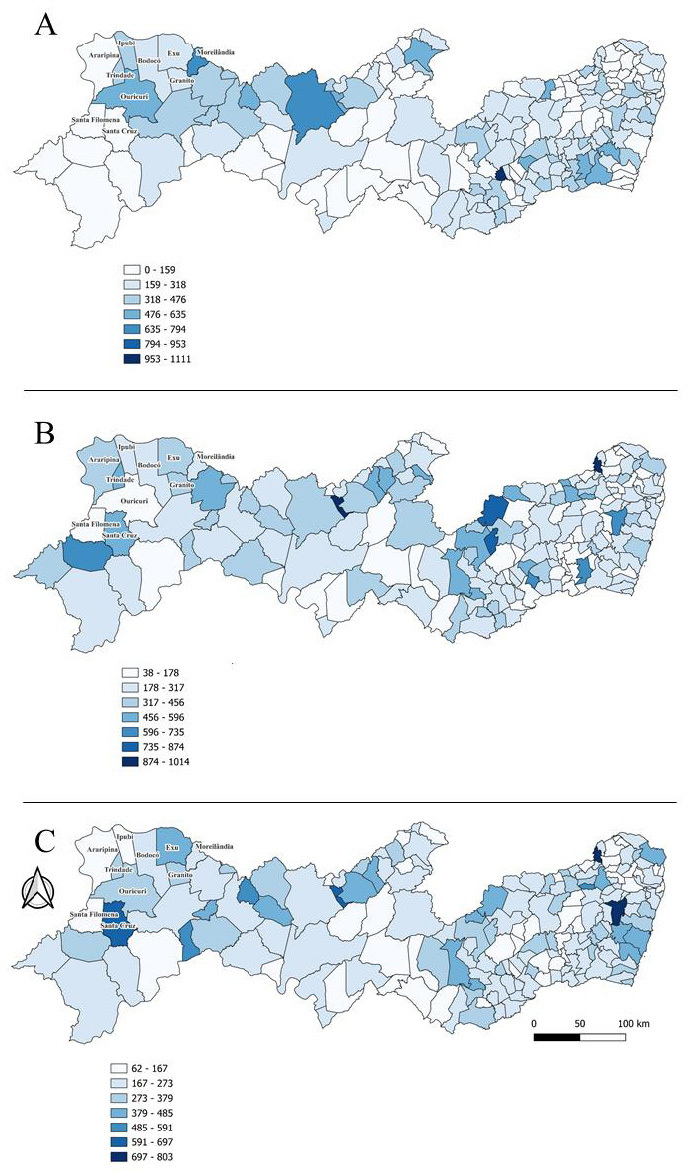
Prevalence of pneumonia cases in Pernambuco, with data for 2008 (A), 2019 (B) and the average between 2008 and 2019 (C), totaling 278,738 cases reported in the study period (2008-2019)

Note: The analyses considered the municipalities within the gypsum hub in Sertão do Araripe (Araripina, Bodocó, Exu, Granito, Ipubi, Moreilândia, Ouricuri, Santa Cruz, Santa Filomena, and Trindade), and the municipalities outside the gypsum hub (the remaining 175 municipalities of Pernambuco).

In assessing the prevalence of respiratory diseases between municipalities in the gypsum hub and those outside, analysis of variance of repeated measures revealed a statistical difference. The F-test yielded values of 28.94 (p-value<0.050) for municipalities outside the gypsum hub and 2.46 (p-value<0.050) for those within the hub. Among the economically active population (15–59 years), variability was observed between the ten municipalities of the Sertão do Araripe and the remaining 175 municipalities of Pernambuco, especially in 2010 and 2011, when elevated prevalence rates of respiratory diseases were recorded, reaching 187.80 and 157.10 cases per 100,000 inhabitants, respectively (Figure 2). There was an observable evolution of pneumonia in Pernambuco from 2008 to 2019 (Figure 3).

Pearson’s correlation analysis revealed a strong positive relationship between the population size in the gypsum production hub region and gross domestic product (r 0.95; p-value<0.050), as well as between respiratory diseases and the hospital bed occupancy rate (r 0.24; p-value<0.050). However, the correlation between per capita public health expenditures and respiratory diseases was low and negative. There was also a positive correlation between respiratory diseases and hospital bed occupancy rates (r 0.24; p-value<0.050). Additionally, a negative correlation was found between the respiratory disease rate and health expenditures per capita.

Among the respiratory diseases analyzed, only pneumonia showed a statistically significant difference. Given this, a multiple linear regression analysis was conducted (Table 1), and the results showed an association between the presence of the gypsum production hub and pneumonia rates in all five models tested.

**Table 1:** Multiple linear regression analysis of pneumonia rates. Pernambuco, 2008-2019 (n=430,305)

Models tested										
Pneumonia rate^a^	1	p-value	2	p-value	3	p-value	4	p-value	5	p-value
Gypsum hub	0.27; 0.07^s,b^	<0.001	0.28; 0.07^s^	<0.001	0.28; 0.07^s^	<0.001	0.29; 0.07^s^	<0.001	0.28; 0.07^s^	<0.001
Population size	-		0.12; 0.02^s^	<0.001	0.09; 0.08	0.275	0.09; 0.08	0.246	0.10; 0.08	0.217
Gross domestic product per capita			-		0.02; 0.06	0.699	0.02; 0.06	0.688	<0.01; 0.06	0.935
per capita public health expenditures	-		-		-		0.07; 0.02	0.001	0.08; 0.02	0.001
Hospital bed rate	-		-		-		-		0.25; 0.40^s^	<0.001

^a^Dependent variable: hospitalization rate for pneumonia (per 100,000 inhabitants); ^b^S: statistical difference at the 5% level (p-value<0.050). The values are presented as estimates of the coefficient and the standard error. All models use log-transformation of continuous independent variables; s: represents the data that shows statistical difference.

In Model 1, which only considered variables belonging to the gypsum hub, there was evidence of a statistical difference, with a coefficient of 0.28 (0.07; p-value < 0.001). It indicated that, on average, municipalities in the gypsum hub had approximately 27.0% higher pneumonia hospitalization rates compared to other municipalities in Pernambuco. The R^2^ value for this model was 0.01, indicating that the included variables explained 1.0% of the variability in prevalence rate.

With the inclusion of the population size variable in Model 2, the coefficient for the gypsum hub remained statistically significant at 0.28 (0.07; p-value<0.001). With the logarithm of population size remaining constant, the presence of the gypsum hub continued to be associated with a positive impact and a statistical difference in the rate of hospital admissions for pneumonia. Nevertheless, the coefficient related to the impact of the logarithm of population size did not show statistical significance. The R^2^ increased to 0.03, indicating that adding the population size variable to the model improved the model’s ability to explain the variability in prevalence.

In Model 3, which included the logarithm of gross domestic product, the gypsum hub variable remained statistically significant (coefficient 0.29; p-value<0.001). Nonetheless, there was no evidence of association for population size (p-value 0.275) and gross domestic product (p-value 0.699). Model 4, which included the logarithm of per capita public health expenditure, revealed an association with variables belonging to the gypsum hub (coefficient 0.30, p-value<0.001) and per capita public health expenditure (coefficient 0.08; p-value 0.001).

In Model 5, after including the logarithm of the hospital bed rate, statistically significant associations were found for the variables belonging to the gypsum production hub (coefficient 0.28; p-value<0.001), per capita public health expenditures (coefficient 0.08; p-value 0.001), and hospital bed rate (coefficient 0.25; p-value<0.001). Variables such as population size (p-value 0.217) and gross domestic product per capita (p-value 0.935) showed no evidence of association. The R^2^ value increased to 0.08, suggesting that the model explained 8.0% of the variability in prevalence rate. These results suggest that the presence of the gypsum production hub is associated with increased pneumonia prevalence, even after adjusting for independent variables such as population size, gross domestic product, per capita public health expenditures, and hospital bed rate.

## Discussion

This study found a higher prevalence of respiratory diseases in municipalities within the gypsum production hub of Pernambuco compared to those located outside this region. This finding is consistent with data on industrial exposure, which shows greater susceptibility among children under 5 years of age and older adults over 80 years old (15–18). This highlights the vulnerability of these age groups to environmental and occupational factors.

One of the main limitations was aggregation bias, also referred to as ecological fallacy, which is inherent to studies based on aggregated data. This bias occurs when causal relationships are inferred based on population-level observations, given that exposures and health conditions are not directly assessed at the individual level (19,20). Furthermore, the reliance on secondary data represented a limitation, as these data may be subject to underreporting, particularly in conditions such as pneumoconiosis (9,21). 

This study provides a relevant contribution by identifying a significant association between the gypsum production hub region and the prevalence of respiratory diseases, supporting previous studies that link exposure to air pollutants and particulate matter to respiratory morbidity and mortality (1–7). Such associations are also observed in populations exposed to household air pollution in low- and middle-income countries, underscoring vulnerabilities associated with socioeconomic and environmental factors (22–24). 

Evidence suggests an association between exposure to silica dust—present in mining activities—and the incidence of lung cancer and other respiratory diseases in workers, which reinforces the occupational impact identified in this study (25). In contrast to studies conducted in urban areas, where pollution of vehicular and industrial origin predominates (26,27), the data in this study reflected the influence of gypsum industry activities on air quality in the Sertão do Araripe region. Replacing firewood with natural gas in the gypsum industry may represent an effective strategy to reduce the emission of toxic pollutants, while also promoting economic and environmental sustainability (28).

Based on these findings, it is suggested that gypsum production activity is associated with the prevalence of respiratory diseases in the studied region. The implementation of public policies to mitigate the impact of industrial pollution and improve working conditions in the gypsum sector may contribute to reducing respiratory morbidity rates while also promoting health and sustainability in the region. To strengthen causal inference between occupational exposure in the gypsum industry and respiratory illness, observational studies with an individual-level approach, such as cohort studies, are needed. These would allow for the control of confounding variables and the analysis of the progression of respiratory conditions over time.

## Data Availability

The data used in the research are available in the repository: https://drive.google.com/drive/folders/10-If1KihCLCp8dqn2WxT6SXqtwezkbzW?usp-valor=sharing.
