# Supplementary material for: High prevalence of respiratory diseases: a population-based ecological study, Sertão do Araripe, 2008-2019
Source: Epidemiol Serv Saude. 2025 Sep 15;34:e20240519. doi: 10.1590/S2237-96222025v34e20240519.en (PMC12435955; doi:10.1590/S2237-96222025v34e20240519.en)
Supplement: Translation

# Translation: Alta prevalência de doenças respiratórias: estudo ecológico de base populacional, Sertão do Araripe, 2008-2019

**DOI:** 10.1590/S2237-96222025v34e20240519.pt

**Authors:** José Rafael Soares da Silva, Rodrigo Gomes de Arruda, Bárbara de Oliveira Silva, Erika da Silva Bezerra de Menezes, Cinthia Martins Menino Diniz, João Pedro Alves Gomes, Breno Caldas de Araújo, Moacyr Jesus Barreto de Melo Rêgo, Maira Galdino da Rocha Pitta, Michelly Cristiny Pereira

**Affiliations:** 1 Universidade Federal de Pernambuco, Núcleo de Pesquisa em Inovação Terapêutica Suely Galdino, Recife, PE, Brasil; 2 Universidade Federal de Pernambuco, Departamento de Economia, Recife, PE, Brasil

**Keywords:** Doenças das Vias Respiratórias, Pneumonia, Saúde Ocupacional, Taxa de Mortalidade, Taxa de Prevalência

## Abstract

**Objetivo:**

Investigar a prevalência e a mortalidade das doenças respiratórias, entre 2008 e 2019, dos municípios com atividade gesseira na região do Sertão do Araripe, Pernambuco.

**Métodos:**

Tratou-se de estudo ecológico, de base populacional, com dados extraídos dos sistemas de informações hospitalares e de mortalidade, comparando 10 municípios do polo gesseiro aos demais municípios de Pernambuco sem atividade gesseira. As taxas de prevalência e mortalidade foram calculadas por 100 mil e 1 mil habitantes e apresentadas por média (desvio-padrão), para doença pulmonar obstrutiva crônica, asma, bronquite aguda e pneumonia. Testes de correlação de Pearson, U de Mann-Whitney e regressão linear múltipla foram realizados para avaliar a relação entre a atividade gesseira e as doenças respiratórias.

**Resultados:**

No período analisado, foram registrados 17.630 casos de doenças respiratórias, com prevalência média de 454,74 (147,16) por 100 mil habitantes e mortalidade de 0,34 (0,08) por 1 mil habitantes. A pneumonia respondeu por 12.680 internações, com prevalência de 431,20 (173,04) por 100 mil habitantes, sendo mais frequente em Ouricuri (543,08), Ipubi (409,93) e Moreilândia (404,80). Observou-se maior prevalência de pneumonia nos municípios do polo gesseiro (p-valor 0,007). Verificou-se correlação positiva entre doenças respiratórias e taxa de ocupação de leitos hospitalares (r=0,24; p-valor<0,050), e negativa com os gastos públicos em saúde per capita. Houve associação significativa entre a presença da indústria gesseira e a pneumonia (p-valor<0,001).

**Conclusão:**

A atividade gesseira está associada à prevalência de doenças respiratórias na região, com destaque para os elevados índices de pneumonia.

**Aspectos éticos**

Esta pesquisa utilizou bancos de dados de domínio público e anonimizados.:

## Introdução

O estado de Pernambuco detém aproximadamente 18,0% das reservas brasileiras de gipsita e responde por 97,0% da produção nacional desse mineral (1). A extração e o beneficiamento concentram-se predominantemente no Sertão do Araripe, abrangendo os municípios de Araripina, Bodocó, Exu, Granito, Ipubi, Moreilândia, Ouricuri, Santa Cruz, Santa Filomena e Trindade (2).

A indústria gesseira é fundamental para o crescimento econômico, pois promove a geração de empregos e contribui para o desenvolvimento socioeconômico da região. No entanto, os processos de calcinação e extração do gesso apresentam riscos potenciais à saúde respiratória da população local (3). A exposição prolongada a agentes presentes em empresas de mineração ou construção civil, como a sílica (poeira de gesso) e a fumaça (queima de biomassa), pode aumentar o risco de desenvolvimento de processos inflamatórios e infecciosos nos pulmões (4), além de diversas doenças do trato respiratório, como silicose (5), asma (6), bronquite, pneumonia (7), doença pulmonar obstrutiva crônica (8) e pneumoconiose (7,9) e câncer de pulmão (10). 

Devido à limitação de estudos que abordem aspectos epidemiológicos de doenças respiratórias no Sertão do Araripe, bem como da necessidade de implementar políticas de saúde pública eficazes, este estudo teve como objetivo investigar a prevalência e a mortalidade das doenças respiratórias, entre 2008 e 2019, dos municípios que apresentavam atividade gesseira nessa região.

## Métodos

### Delineamento e tamanho do estudo

Tratou-se de estudo ecológico de base populacional, conduzido entre 2008 e 2019, que analisou dados referentes a 185 municípios de Pernambuco, sendo 10 municípios da região do Sertão do Araripe e 175 municípios restantes, além do arquipélago de Fernando de Noronha. O estudo abrangeu dados de internações e óbitos relacionados a doenças respiratórias, incluindo pneumoconiose, bronquite aguda, pneumonia, asma e doença pulmonar obstrutiva crônica, associadas à exposição à poeira de gesso, afetando tanto a população geral quanto a população economicamente ativa (indivíduos entre 15-59 anos).

### Contexto e controle de viés

O período selecionado foi estabelecido para avaliar exclusivamente os dados prévios à pandemia da covid-19. A exclusão dos dados subsequentes a esse intervalo justifica-se pelo aumento exponencial no número de casos e óbitos atribuídos ao novo coronavírus no Brasil a partir de 2020 (11), o que poderia introduzir vieses nas análises realizadas.

### Local e população do estudo

Pernambuco possui, de acordo com o Censo Demográfico de 2022, uma população estimada em 9.058.931 habitantes, com densidade demográfica de 92,37 habitantes por km^2^. O estado ocupa a 19ª posição em extensão territorial no Brasil, com área de 98.067,877 km^2^, e, em 2021, apresentava o 15º pior índice de desenvolvimento humano do país, abaixo da média nacional (12).

Foram selecionados dados de internações e óbitos como parâmetros comparativos entre as regiões previamente mencionadas.

### Variáveis do estudo

As variáveis independentes selecionadas neste estudo foram: presença do polo gesseiro; tamanho da população; produto interno bruto per capita (com valores ajustados a preços de 2010); despesas em saúde pública per capita; e taxa de leitos hospitalares. 

Os dados das variáveis tamanho da população e produto interno bruto per capita (com valores ajustados a preços de 2010) foram obtidos a partir do Instituto Brasileiro de Geografia e Estatística (disponíveis em: https://cidades.ibge.gov.br/). Os dados das variáveis despesas em saúde pública per capita e taxa de leitos hospitalares foram obtidos através do Sistema de Informações Hospitalares do Departamento de Informática do Sistema Único de Saúde (disponíveis em: https://datasus.saude.gov.br/acesso-a-informacao/morbidade-hospitalar-do-sus-sih-sus/). Os dados foram analisados com o intuito de avaliar possíveis correlações e influências sobre as taxas de prevalência das doenças respiratórias. 

### Sistemas de informações utilizadas 

Foram analisados os dados de morbidade hospitalar provenientes do Sistema de Informações Hospitalares e Sistema de Informações sobre Mortalidade, disponíveis no Departamento de Informática do Sistema Único de Saúde, por local de residência em Pernambuco (disponíveis em: http://datasus.saude.gov.br/acesso-a-informacao/morbidade-hospitalar-do-sus-sih-sus/). As taxas de prevalência e mortalidade foram calculadas com base nos dados populacionais do Instituto Brasileiro de Geografia e Estatística – dados populacionais com base no Censo Demográfico de 2022 (disponíveis em: https://www.ibge.gov.br/). 

A confirmação das atividades econômicas relacionadas à produção gesseira foi realizada por meio da Plataforma de Relação Anual de Informações Sociais, referente à produção gesseira no período 2008-2019 (disponível em: https://bi.mte.gov.br/bgcaged/). A consulta considerou as seguintes subclasses da Classificação Nacional de Atividades Econômicas 2.0: (i) extração de gesso e caulim, abrangendo a mineração de matérias-primas para produção; (ii) fabricação de artefatos de gesso e materiais similares, destinados à construção civil; (iii) fabricação de cal e gesso, incluindo os processos industriais de calcinação; e (iv) obras de acabamento em gesso e estuque, representando serviços especializados na aplicação de revestimentos e molduras em edificações. Esses dados não necessitaram de aprovação de comitê de ética por serem de domínio público.

### Análise estatística

Os dados foram organizados em planilhas no Microsoft Excel 2010 e analisados com o software STATA 17.0 MP. Foram calculadas medidas descritivas, como médias, medianas e desvios-padrão, e construídos gráficos do tipo diagrama de caixa para ilustrar a distribuição da prevalência e mortalidade por doenças respiratórias. A comparação das taxas de prevalência entre diferentes faixas etárias nos municípios pernambucanos foi realizada por meio do teste de hipótese de U de Mann-Whitney, apropriado para comparação e distribuição de dados sem suposições sobre distribuições de probabilidade.

A análise das tendências temporais nas taxas de prevalência foi conduzida pela análise de variância de medidas repetidas (ANOVA), com cálculo do teste F, permitindo comparar a evolução das taxas entre municípios com e sem atividade gesseira. A correlação de Pearson foi realizada para identificar associações entre as doenças respiratórias, variáveis socioeconômicas e infraestrutura em saúde por meio de análise bivariável. Posteriormente, um modelo de regressão linear múltipla foi estimado pelo método dos mínimos quadrados ordinários, com seleção de variáveis do tipo forward (13,14), e representado pela equação: (taxa de doença)= B^0^+B^1^ (polo gesseiro)+B^2^ (tamanho da população)+B^3^ (produto interno bruto per capita)+B^4^ (despesa pública em saúde per capita)+B^5^ (taxa de leitos)+ε.

O coeficiente de determinação (R^2^) foi utilizado para mensurar a variabilidade explicada, e a análise de resíduos avaliou a adequação do modelo (Figura suplementar 1).

As análises foram centradas nos 10 municípios do Sertão do Araripe, devido à importância da indústria gesseira na região. Para testar a robustez dos efeitos, variáveis independentes foram introduzidas isoladamente em modelos distintos, permitindo avaliar a estabilidade e a significância estatística dos coeficientes, especialmente da variável presença do polo gesseiro. A confecção dos mapas temáticos de prevalência foi feita com o QGIS 3.30.2 e utilizou sete gradientes de cor para representar a distribuição espacial das doenças respiratórias nos municípios analisados.

### Acesso aos dados e métodos de limpeza

Foram extraídos das bases de dados os registros de internações e óbitos por doenças respiratórias, incluindo pneumoconiose (Classificação Estatística Internacional de Doenças e Problemas Relacionados com a Saúde [CID] J63), bronquite aguda (CID J20), pneumonia (CID J18), asma (CID J45) e doença pulmonar obstrutiva crônica (CID J44), associadas à exposição ocupacional e ambiental à poeira de gesso (7). Adicionalmente, analisou-se se havia diferença significativa nas taxas dessas doenças entre a população economicamente ativa (indivíduos entre 15-59 anos) residente nos 10 municípios do sertão do Araripe em comparação com os demais 175 municípios. 

### Pareamento dos dados

O pareamento foi realizado com base em variáveis socioeconômicas, incluindo tamanho da população, produto interno bruto per capita, despesas em saúde pública per capita e taxa de leitos hospitalares dos 185 municípios de Pernambuco. Essas variáveis foram controladas para garantir que as diferenças observadas nas taxas de prevalência e mortalidade por doenças respiratórias fossem atribuídas, principalmente, à presença do polo gesseiro. Considerou-se diferença estatística quando o p-valor foi inferior a 0,05.

## Resultados

A região do Sertão do Araripe registrou 17.630 casos de doenças respiratórias, incluindo asma, bronquite aguda, doença pulmonar obstrutiva crônica e pneumonia entre 2008 e 2019. A taxa média de prevalência foi 454,74 (147,16) casos por 100 mil habitantes, com crianças de 1-4 anos apresentando a maior média de 984,04 (384,14) casos, seguidas por idosos acima de 80 anos, com média de 882,95 (270,15) casos. A taxa média de mortalidade foi 0,34 (0,08) casos por 1 mil habitantes.

Houve 1.474 internações no Sertão do Araripe no que diz respeito à asma, onde apresentou taxa média (desvio-padrão) de prevalência de 52,26 (70,06) casos por 100 mil habitantes, com as maiores prevalências entre 2008 e 2010. Moreilândia, Ipubi e Granito apresentaram as maiores taxas, sendo elas: 246,67; 63,77 e 44,25. A população mais afetada pela doença foram crianças de 1-4 anos de idade com média de 124 (122,15) casos, crianças de 5-9 anos com média de 98,38 (135,95) casos e idosos de 60-69 anos com média de 66,83 (117,46) casos. As maiores taxas de mortalidade por 1 mil habitantes foram observadas em Granito, Exu e Trindade (0,02; 0,01 e 0,01).

Para a bronquite aguda, a taxa média de prevalência para a região foi 14,20 (16,02) casos por 100 mil habitantes. Araripina, Ipubi e Ouricuri apresentaram as maiores taxas, com 58,17; 15,70 e 12,51 casos por 100 mil habitantes. O ano de 2011 apresentou a maior média de prevalência para a região com 24,54 casos para 100 mil habitantes. As faixas etárias mais atingidas pela doença, em relação ao cálculo de prevalência, foram bebês entre 0-1 ano com 72,77 (105,66) casos, crianças de 1-4 anos com 39,81 (76,86) casos e crianças de 5-9 anos com 10,47 (31,42) casos. Apenas Araripina e Ouricuri registraram óbitos, com taxa de mortalidade inferior a 0,01 casos por 1 mil habitantes em ambos os municípios.

Foram identificadas 2.578 internações em relação à doença pulmonar obstrutiva crônica, com taxa média de prevalência para a região de 55,11 (49,72) casos por 100 mil habitantes. Houve aumentos e declínios intermitentes ao longo dos anos, sendo Araripina e Granito os municípios mais incidentes, com 153,95 (92,33) e 142,33 (168,99) casos. As faixas etárias mais atingidas pela doença na região foram entre indivíduos de 70-79 anos, com 123,68 (76,97) casos por 100 mil habitantes. As maiores médias das taxas de mortalidade por doença pulmonar obstrutiva crônica ficaram com Moreilândia (0,158 casos por 1 mil habitantes), Santa Filomena (0,11 casos por 1 mil habitantes) e Bodocó (0,11 casos por 1 mil habitantes).

Houve 12.680 casos de internações referentes à pneumonia na região, com alta prevalência em 2011 com 451,155 (294,70) casos por 100 mil habitantes, diminuição em 2013 e novos aumentos dos casos entre 2017, com 389,26 (103,11) casos, e 2019, com 431,20 (173,04) casos por 100 mil habitantes. Essa foi a única doença com prevalência significativamente maior entre os 10 municípios do Sertão do Araripe comparado aos 175 municípios de Pernambuco fora do polo. As maiores taxas de prevalência foram em Ouricuri, Ipubi e Moreilândia, com taxas de 543,08 (267,11); 409,93 (77,31) e 404,80 (305,01). Crianças entre 1-4 anos de idade apresentaram as maiores taxas de prevalência, com 758,46 (394,31) casos, e idosos com mais de 80 anos de idade, com 697,61 (246,46) casos por 100 mil habitantes. As maiores médias das taxas de mortalidade de pneumonia foram em Bodocó, Exu e Moreilândia, com 0,26; 0,25 e 0,25 casos por 1 mil habitantes.

Não foram observadas diferenças estatisticamente significativas nas taxas de prevalência e mortalidade da população economicamente ativa quando comparadas a cada doença individualmente, exceto no caso da pneumonia. Quanto à pneumonia, a mediana de prevalência foi significativamente maior nos municípios do polo gesseiro quando comparada ao polo não gesseiro (Figura 1).

**Figura 1:** Mediana da taxa de prevalência de doenças respiratórias, com dados para a população geral (A), crianças menores de 14 anos (B), população economicamente ativa de 15-59 anos (C) e idosos com 60 anos ou mais (D), nos 10 municípios do Sertão do Araripe em comparação com os 175 municípios de Pernambuco, 2008-2019, com 430.305 casos incluídos

A mediana no polo gesseiro foi 357,20 (n=10), enquanto foi 213,20 (n=175) no polo não gesseiro, havendo diferença estatística (U 1.571,0; p-valor 0,007). Em relação à população com idade inferior a 14 anos, observou-se também a prevalência mais elevada de pneumonia nos municípios do polo gesseiro, que foi 138,70 (n=10), em comparação ao polo não gesseiro (mediana 83,08), com evidência de diferença estatística (U 1.580,0; p-valor 0,040). Para a população economicamente ativa (15-59 anos), a prevalência foi maior no polo gesseiro (mediana 67,87) em relação ao não gesseiro (mediana 38,90; U 1.575,0; p-valor<0,001). Entre os indivíduos idosos, a prevalência também foi mais elevada no polo gesseiro (mediana 124,4) quando comparada ao não gesseiro (mediana 82,18; U 1.387,0; p-valor 0,014). Os casos de internação por pneumoconiose foram raros, com apenas um caso em Araripina e outro caso em Bodocó, não havendo óbitos.

Na avaliação das taxas de prevalência das doenças respiratórias entre os municípios do polo gesseiro e os de fora do polo, a análise de variância de medidas repetidas revelou que houve diferença estatística. O teste F indicou valores de 28,94 (p-valor<0,050) para os municípios fora do polo gesseiro e 2,46 (p-valor<0,050) para aqueles do polo gesseiro. Na população economicamente ativa (faixa etária 15-59 anos), observou-se a variabilidade entre os 10 municípios do Sertão do Araripe e os 175 restantes de Pernambuco, especialmente durante 2010 e 2011, quando foram registradas taxas de prevalência elevadas para doenças respiratórias, atingindo 187,80 e 157,10 casos por 100 mil habitantes (Figura 2). Observou-se a evolução da pneumonia em Pernambuco no período 2008-2019 (Figura 3).

**Figura 2:** Média da taxa de prevalência por doenças respiratórias, incluindo asma, bronquite aguda, doença pulmonar obstrutiva crônica e pneumonia, para a população geral (A), população economicamente ativa de 15-59 anos com doenças respiratórias (B) e a população economicamente ativa de 15-59 anos com pneumonia (C), nos 10 municípios do Sertão do Araripe e nos 175 municípios restantes de Pernambuco, 2008-2019, com 430.305 casos incluídos

Nota: As análises consideraram os municípios do polo gesseiro, composto pelos 10 municípios do Sertão do Araripe (Araripina, Bodocó, Exu, Granito, Ipubi, Moreilândia, Ouricuri, Santa Cruz, Santa Filomena e Trindade), e os municípios fora do polo gesseiro (175 municípios restantes de Pernambuco).

**Figura 3:** Prevalência dos casos de pneumonia em Pernambuco, com dados para 2008 (A), 2019 (B) e a média entre 2008 e 2019 (C), totalizando 278.738 casos notificados no período estudado (2008-2019)

A análise de correlação de Pearson revelou a relação alta e positiva entre o tamanho da população da região do polo gesseiro e o produto interno bruto (r=0,95; p-valor<0,050) e entre as doenças respiratórias e a taxa de ocupação de leitos (r=0,24; p-valor<0,050), no entanto a correlação entre a taxa de despesas em saúde per capita e as doenças respiratórias foi baixa e negativa. Também foi observada a correlação positiva entre as doenças respiratórias e as taxas de leito ocupadas (r=0,24; p-valor<0,050). Ademais, identificou-se a correlação negativa entre a taxa de doenças respiratórias e os gastos per capita com saúde.

Entre as doenças respiratórias analisadas, apenas a pneumonia apresentou diferença estatisticamente significativa. Diante disso, foi conduzida análise de regressão linear múltipla (Tabela 1), e os resultados mostraram associação entre a presença do polo gesseiro e as taxas de pneumonia em todos os cinco modelos testados.

**Tabela 1:** Análise da regressão linear múltipla da taxa de pneumonia. Pernambuco, 2008-2019 (n=430.305)

Modelos testados										
Taxa de pneumonia^a^	1	p-valor	2	p-valor	3	p-valor	4	p-valor	5	p-valor
Polo gesseiro	0,27; 0,07^s,b^	<0,001	0,28; 0,07^s^	<0,001	0,28; 0,07^s^	<0,001	0,29; 0,07^s^	r<0,001	0,28; 0,07^s^	<0,001
Tamanho da população	-		0,12; 0,02^s^	<0,001	0,09; 0,08	0,275	0,09; 0,08	0,246	0,10; 0,08	0,217
Produto interno bruto per capita			-		0,02; 0,06	0,699	0,02; 0,06	0,688	<0,01; 0,06	0,935
Despesa em saúde per capita	-		-		-		0,07; 0,02	0,001	0,08; 0,02	0,001
Taxa de leitos	-		-		-		-		0,25; 0,40^s^	<0,001

^a^Variável dependente: taxa de internações por pneumonia (por 100 mil habitantes); ^b^S: diferença estatística ao nível de 5% (p-valor<0,050). Os valores são apresentados como estimativa do coeficiente e erro padrão. Todos os modelos utilizam log-transformação das variáveis independentes contínuas; ^s^: representação dos dados que apresentaram diferença estatística.

No modelo 1, que considerou apenas a variável pertencer ao polo gesseiro, houve evidência de diferença estatística, com coeficiente de 0,28 (0,07; p-valor<0,001). Isso indicou que, em média, os municípios do polo gesseiro tinham taxa de internações por pneumonia cerca de 27,0% maior em comparação ao demais municípios de Pernambuco. O valor de R^2^ para este modelo foi 0,01, indicando que 1,0% da variabilidade na taxa de prevalência foi explicada pelas variáveis incluídas.

Com a inclusão da variável tamanho da população no modelo 2, observou-se que o coeficiente para polo gesseiro permaneceu com diferença estatística em 0,28 (0,07; p-valor<0,001). Mantendo-se constante o logaritmo do tamanho da população, a presença do polo gesseiro continuou associada ao impacto positivo e à diferença estatística na taxa de internações por pneumonia. Entretanto, o coeficiente relacionado ao impacto do logaritmo do tamanho da população não apresentou diferença estatística. O valor de R^2^ aumentou para 0,03, indicando que a adição da variável tamanho da população ao modelo contribuiu para explicar mais variabilidade na taxa da prevalência.

No Modelo 3, que incluiu o logaritmo do produto interno bruto, a variável pertencer ao polo gesseiro permaneceu com diferença estatística (coeficiente 0,29; p-valor<0,001). Contudo, não houve evidência de associação para o tamanho da população (p-valor 0,275) e produto interno bruto (p-valor 0,699). O Modelo 4, que incluiu o logaritmo das despesas em saúde per capita, demonstrou associação com as variáveis pertencentes ao polo gesseiro (coeficiente 0,30, p-valor<0,001) e despesas em saúde per capita (coeficiente 0,08; p-valor 0,001).

No Modelo 5, com a inclusão do logaritmo da taxa de leitos hospitalares, a variável pertencer ao polo gesseiro manteve associação (coeficiente 0,28; p-valor<0,001), assim como despesas em saúde per capita (coeficiente 0,08; p-valor 0,001) e taxa de leitos hospitalares (coeficiente 0,25; p-valor<0,001). As variáveis como tamanho da população (p-valor 0,217) e produto interno bruto per capita (p-valor 0,935) não apresentaram evidência de associação. O valor de R^2^ aumentou para 0,08, o que sugere que o modelo explica 8,0% da variabilidade na taxa de prevalência. Esses resultados sugeriram que a presença do polo gesseiro esteja associada a um aumento na prevalência de pneumonia, mesmo após o ajuste por variáveis independentes como tamanho da população, produto interno bruto, despesas em saúde per capita e taxa de leitos hospitalares.

## Discussão

Este estudo identificou a maior prevalência de doenças respiratórias nos municípios pernambucanos do polo gesseiro, em comparação àqueles localizados fora dessa região. Esse achado é consistente com dados sobre a exposição industrial, nos quais a maior suscetibilidade foi observada em crianças menores de 5 anos e idosos acima de 80 anos (15-18). Isso ressalta a vulnerabilidade desses grupos etários a fatores ambientais e ocupacionais.

Entre as limitações, destacou-se o viés de agregação, também conhecido como falácia ecológica, intrínseco a estudos que utilizam dados agregados. Esse viés ocorre ao inferir relações causais com base em observações populacionais, uma vez que exposições e condições de saúde não são diretamente avaliadas em nível individual (19,20). Além disso, a dependência de dados secundários representou uma limitação, visto que esses dados podem apresentar subnotificação, especialmente em condições como a pneumoconiose (9,21). 

Este estudo apresentou contribuição relevante ao identificar associação significativa entre a região do polo gesseiro e a prevalência de doenças respiratórias, corroborando estudos que relacionam a exposição a poluentes atmosféricos e material particulado à morbimortalidade respiratória (1-7). Tal relação é semelhante em populações expostas a poluentes domiciliares em países de baixa e média renda, destacando vulnerabilidades associadas a fatores socioeconômicos e ambientais (22-24). 

Evidências sugerem associação entre a exposição à poeira de sílica, presente em atividades mineradoras, e a incidência de câncer de pulmão e outras doenças respiratórias em trabalhadores, o que reforça o impacto ocupacional identificado neste estudo (25). Em contraste com estudos realizados em regiões urbanas, onde a poluição de origem veicular e industrial é predominante (26,27), os dados deste estudo refletiram a influência das atividades do setor gesseiro na qualidade do ar no Sertão do Araripe. A substituição da lenha por gás natural na indústria do gesso pode representar estratégia eficaz para mitigar a emissão de poluentes tóxicos, além de favorecer a sustentabilidade econômica e ambiental (28).

Com base nestes resultados, sugere-se que a atividade gesseira está associada à prevalência de doenças respiratórias na região estudada. A adoção de políticas públicas para mitigar o impacto da poluição industrial e melhorar as condições laborais no setor gesseiro pode contribuir para a redução dos índices de morbidade respiratória, além de favorecer a promoção da saúde e a sustentabilidade da região. Para o fortalecimento da inferência causal entre a exposição ocupacional no setor gesseiro e o adoecimento respiratório, são necessários estudos observacionais com abordagem individual, como estudos de coorte, que permitam controlar variáveis de confusão e analisar a progressão das condições respiratórias ao longo do tempo.

## Data Availability

Os dados utilizados na pesquisa estão disponíveis no repositório: https://drive.google.com/drive/folders/10-If1KihCLCp8dqn2WxT6SXqtwezkbzW?usp-valor=sharing.
